# A physical activity, nutrition and oral health intervention in nursery settings: process evaluation of the NAP SACC UK feasibility cluster RCT

**DOI:** 10.1186/s12889-019-7102-9

**Published:** 2019-07-03

**Authors:** Rebecca Langford, Russell Jago, James White, Laurence Moore, Angeliki Papadaki, William Hollingworth, Chris Metcalfe, Dianne Ward, Rona Campbell, Sian Wells, Ruth Kipping

**Affiliations:** 1 DECIPHer, Bristol Medical School, University of Bristol, Oakfield House, Oakfield Grove, Bristol, BS8 2BN UK; 2 Centre for Exercise, Nutrition and Health Sciences, School for Policy Studies, University of Bristol, Social Science Complex, 8 Priory Rd, Bristol, BS8 1TZ UK; 3 Centre for Trials Research, DECIPHer, College of Biomedical and Life Sciences, Cardiff University, 4th Floor Health Park, Cardiff, CF14 4YS UK; 4 Institute of Health & Wellbeing, MRC/CSO Social and Public Health Sciences Unit, University of Glasgow, 200 Renfield Street, Glasgow, G2 3AX UK; 5 Bristol Medical School, University of Bristol, Canynge Hall, 39 Whatley Rd, Bristol, BS8 2PS UK; 6 Department of Nutrition, Gillings School of Global Public Health, Centre for Health promotion and Disease Prevention, University of North Carolina, 1700 M.L.K. Jr Blvd #7426, Chapel Hill, NC 27514 USA; 7 DECIPHer, Bristol Medical School, University of Bristol, Canynge Hall, 39 Whatley Rd, Bristol, BS8 2PS UK

**Keywords:** Obesity, Physical activity, Nutrition, Oral health, Child care, Preschool children, Intervention, Process evaluation, Feasibility

## Abstract

**Background:**

The nutrition and physical activity self-assessment for childcare (NAP SACC) intervention has demonstrated effectiveness in the USA. A feasibility randomised controlled trial was conducted in England to adapt the intervention to the UK context. An embedded process evaluation focused on three key questions. 1. Was it feasible and acceptable to implement the intervention as planned? 2. How did the intervention affect staff and parent mediators? 3. Were the trial design and methods acceptable?

**Methods:**

Twelve nurseries in south-west England were recruited and randomised to intervention or control. The intervention comprised: NAP SACC UK Partner (Health Visitor) support to nurseries to review practice and policies against best practice, and then set goals to improve physical activity, nutrition and oral health; two staff training workshops; and a web-based parent support element. The process evaluation comprised: observations of Partner training (*n* = 1), Partner/manager meetings (*n* = 5) and staff workshops (*n* = 10); semi-structured interviews with Partners (*n* = 4), managers (*n* = 12), staff (n = 4) and parents (*n* = 20); analysis of self-assessment forms, goal setting forms and Partner logbooks; and assessment of staff and parent knowledge, motivation and self-efficacy mediators.

**Results:**

Overall, NAP SACC UK was feasible to implement and acceptable to nursery staff, managers, Partners and parents. The intervention was implemented as planned in five of the six intervention nurseries. Partners and managers appreciated the opportunity to review and improve nursery practices and valued the relationship forged between them. Staff rated the training workshops highly, despite attending outside of working hours. Most goals set by nurseries were achieved. However, Partners raised concerns about Health Visitors’ capacity to deliver the intervention in any subsequent roll out. Mediator scores improved in all but two areas in intervention staff and parents, with decreases or minimal changes in the control group. The web-based parent element was not well used and should be removed from any subsequent trial. The trial methods were acceptable to managers, staff, Partners and parents.

**Conclusions:**

Implementing and evaluating a physical activity and nutrition intervention in nursery settings is feasible and acceptable. A full RCT of NAP SACC UK (with appropriate modifications) is warranted.

**Trial registration:**

ISRCTN16287377 (10 Apr 2015).

**Electronic supplementary material:**

The online version of this article (10.1186/s12889-019-7102-9) contains supplementary material, which is available to authorized users.

## Background

Childhood obesity is a major public health challenge. Globally, 41 million children under 5 years are overweight or obese, with this figure expected to rise to 70 million by 2025 [1]. Overweight and obesity tracks from childhood to adulthood [2], raising significant health, social and economic implications for the future. Intervening early to help children maintain a healthy weight is critical.

In England, 23% of children entering primary school are already overweight or obese, with higher rates in areas of socio-economic deprivation [3]. Physical activity levels and dietary intake in the majority of children in the UK do not meet current national guidelines. Only 9% of 2–4 year olds in England are physically active for the recommended minimum of three hours per day [4]. Young children in the UK obtain over double the recommended 5% of their total dietary energy intake from free sugars (11.5% for 1.5–3 year olds, 13.5% for 4–10 year olds), with saturated fat intake also higher than the recommended 11% for these groups (14.5% for 1.5–3 year olds, 13% for 4–10 year olds) [5].

Early years childcare settings, (also known as nurseries, day care or pre-schools) are a promising environment in which to deliver scalable health interventions at a population level, with over a third (38%) of UK 3–4 year olds attending private or voluntary nurseries outside of school settings [6]. Recent policy changes in England to increase government-funded childcare for this age group to 30 h per week [7] may increase the time children spend in this setting, and thus their potential to influence children’s physical activity and dietary habits. Although guidelines for physical activity [8] for young children and nutrition [9] in early years settings were published in 2011 and 2012, these are only voluntary. Further, the standards for physical activity and nutritionally-balanced meals set by the UK government, and outlined in the Early Years foundation stage statutory framework are less prescriptive than for schools [10]. While many Local Authorities have developed local programmes to improve the physical activity and nutrition in children in early years settings, few have been rigorously evaluated. In the UK, only four RCTs have been conducted [11–14], and none considered both physical activity and nutrition together.

The Nutrition and Physical Activity Self Assessment for Childcare (NAP SACC) intervention was developed in the United States, and focuses on improving physical activity and nutrition via changes to the nursery’s environment, policies and practices through a process of self-assessment and targeted technical assistance [15]. RCTs of the programme in the USA have demonstrated effectiveness on a number of outcomes including: zBMI; accelerometer-measured physical activity; an environmental audit nutrition score; and nursery staff’s health knowledge [15–17]. It is widely used in nurseries across the US.

Given the urgent need to find effective interventions for early years settings, this study sought to adapt and test the feasibility of implementing NAP SACC in a UK setting. Early work included consultations with key stakeholders (childcare staff, Health Visitors, dieticians and Local Authority public health staff) to develop and adapt the intervention to the UK context. Modifications included tailoring the self-assessment tool to comply with UK guidelines on physical activity, nutrition and statutory childcare standards [9, 18], by including questions on salt intake, breakfast consumption, protein and dairy provision, and puddings and snacks offered. In addition, American foods/brands were replaced with UK alternatives. Two more substantial changes were made. First, an oral health component was included, in response to perceived local need and nationally high levels of child tooth decay. (One in four children in England has tooth decay by the time they start school [19]). Second, recognising that children of this age spend the majority of their time with parents, rather than in formal childcare, a NAP SACC UK web-based home component was developed to involve parents and carers (see [20] for more details).

This paper presents data collected during the process evaluation of a feasibility RCT of NAP SACC UK and aimed to answer the following questions posed by our pre-specified progression criteria and logic model ([21], and Additional file 1): 1. Was it feasible and acceptable to key stakeholders (Partners, nursery managers, staff and parents) to implement NAP SACC UK? 2. How did the intervention affect staff and parent mediators of knowledge, motivation and self-efficacy? 3. Were the trial design and methods acceptable?

## Methods

### Sample and recruitment

Nurseries in south-west England were stratified according to location (Gloucestershire or North Somerset), deprivation (three levels, derived from the Index of Multiple Deprivation) and size (small, < 48 children; large, > 48 children) [20]. Nurseries were randomly selected and invited to take part until 12 eligible nurseries were recruited. Nurseries were eligible if they provided at least one main meal a day and had a minimum of 20 children aged 2–4 years attending for at least 12 h per week. Nurseries were randomised (after baseline data collection) to receive the NAP SACC UK intervention (5 months, from Feb-June 2016), or continue with their usual practice.

### NAP SACC UK intervention

A detailed description of the intervention is provided elsewhere [21]; the logic model for the intervention is provided as an Additional file 1. In short, four local Health Visitors were trained to act as NAP SACC UK Partners. Health Visitors are qualified nurses or midwives who have undertaken further training and qualifications in child health, health promotion, public health and education. We chose to employ two Health Visitors (Partners) in each of the two geographical areas to replicate real world implementation, where this role would be provided by several staff. Further, we wished to test the intervention delivery was not dependent on one individual’s style and to give resilience in the event of staff changes.

Partners supported nurseries to: review current policies and practice around nutrition, physical activity and oral health against 80 items, according to best practice standards; set up to eight goals to be implemented over 5 months; and provided nurseries with on-going support via emails, phone calls and face-to-face meetings. Two staff training workshops on physical activity and nutrition were held for each intervention nursery, delivered by local experts including community dietician, oral health and physical activity specialists. They were employed by NAP SACC UK, with funding for these roles coming North Somerset Council and Gloucestershire County Council. The web-based parent element (NAP SACC at home) was promoted by nurseries to parents via newsletters, flyers and mugs. Parents using the website were asked to complete a ‘Healthy Habits’ questionnaire and set goals to improve physical activity, nutrition or oral health in their children. Parents received texts and/or emails to provide support, further information or to encourage further goal setting.

### Outcome measures

Outcome measures for the trial are reported elsewhere [20] but briefly comprised: an Environment Policy Assessment and Observation (EPAO); child physical activity and sedentary time (accelerometry); dietary outcomes (Child and Diet Evaluation Tool, CADET); and child height and weight to determine zBMI. Additional questionnaires measured staff and parent mediators (knowledge, motivation and self-efficacy), quality of life (PedsQL) and nursery, family and healthcare costs.

### Process evaluation

#### Observations

The trial manager conducted non-participant observations of the Partner training session (*n* = 1), Partner-Manager meetings (*n* = 5) and staff workshops (*n* = 10). Observations were semi-structured, recording pre-specified items (e.g. numbers attending, length of training session, facilitator present) alongside open qualitative observations. Observations focused on: who attended; the format of the training/meeting/workshop; topics covered; elements that worked well/less well; and the behaviour, interest and engagement demonstrated by participants.

#### Semi-structured interviews

All NAP SACC UK Partners (*n* = 4) and nursery managers (*n* = 12) were interviewed, as well as one staff member from intervention nurseries (n = 4: one declined to be interviewed; one nursery did not implement the intervention). Interviews were conducted face-to-face or via telephone, according to participant preference, and lasted approximately one hour. A random sample of parents from 10 of the twelve nurseries (intervention and control) were interviewed via telephone (*n* = 20, participation rate of 21%); interviews lasted between 15 and 30 min. Semi-structured interview guides were used, specific to each stakeholder group and interviews were transcribed verbatim.

#### Document analysis

Self-assessment (Review and Reflect) forms were completed by nursery managers at baseline and follow up and comprised 80 questions assessing nursery environment, policies and practice in relation to four areas: nutrition and oral health; physical activity and play; outdoor play and learning; and screen time. Each question was rated on a scale of one (lowest) to four (best practice). Scores for individual items were averaged to create a summary score for each of the four areas and a mean difference from baseline to follow-up was calculated.

Goals set by nurseries were collated and cross-referenced against follow-up Review and Reflect forms and interview data to assess progress made. NAP SACC UK Partners logged time spent on the study by recording the date, type (phone, email, face-to-face meeting) and duration of contact with each nursery.

#### Mediators

Staff and parent mediators were assessed at baseline and follow up, via questionnaires scoring physical activity and nutrition domains on a five-point scale for self-efficacy (1 = Disagree a lot, 5 = Agree a lot) and motivation (1 = Never, 5 = Always), and via multiple choice knowledge scales. The items and scales were found to have good acceptability, internal consistency and test re-test reliability and are reported elsewhere [22].

#### Analysis

Interview transcripts were entered into NVivo (version 10) and analysed using thematic content analysis [23]. An initial coding framework was developed by RL after careful reading of four transcripts, including both deductive codes derived from research questions and inductive codes emerging from the data. This framework was independently applied to two further transcripts by RL and RK; any discrepancies in coding were discussed and appropriate revisions made. RL applied this framework to all subsequent manuscripts, making revisions as necessary in discussion with RK. Quotes indicate position, nursery and interview number, where applicable (e.g. Manager_N9/4 = Manager, Nursery 9, interview 4).

Observational data were entered into an Excel spreadsheet. Review and Reflect, goal setting forms, and Partner logs were entered into the study REDCap database. Observation and documentary data were compared and contrasted with interview data for cross-checking and to identify confirmatory or contradictory results. Summary descriptive statistics were calculated for Review and Reflect and mediator scores using means and standard deviations by allocation arm (where applicable).

## Results

### Nursery recruitment

In North Somerset, the NAP SACC UK study was discussed with nursery managers at a meeting convened by the local council, and advertised in the Council’s Early Years newsletter. Gloucestershire was included as recruitment site after North Somerset, meaning we were unable to hold a similar meeting in this area. Instead, nurseries were sent a letter from the Council, alongside a project information sheet, and a form to return indicating their willingness to take part. In both areas, the study was called ‘NAP SACC UK’ and nurseries were informed it was based on an American programme, adapted for use in the UK. Nurseries were informed the study was being run the University of Bristol, with intervention costs provided by North Somerset Council and Gloucestershire County Council.

Of the 14 nurseries approached in North Somerset, six (43%) agreed to participate. In Gloucestershire, participation rates were lower, with 25 nurseries approached to achieve the required sample of six settings (participation rate 25%). We were unable to recruit a large nursery in the highest deprivation group in Gloucestershire, but met recruitment targets for all other size/deprivation categories. Nurseries declined to participate in the study because they were too busy, were experiencing staffing or financial issues, were already participating in other initiatives or felt they did not need the intervention. No nurseries were lost to follow-up.

We identified two reasons to explain the difference in participation rate between the two areas. First, there were fewer on-going nursery-based initiatives in North Somerset, with nurseries therefore more willing to participate in a new programme. In Gloucestershire, many nurseries were involved in other health-related initiatives such as the ‘Bristol Standard’ [24] and the ‘Smiles Better’ oral health pilot programmes, both of which overlapped in content with NAP SACC UK. Second, as noted above, Gloucestershire was included as a recruitment site later in the study, resulting in a shorter recruitment period which included the Christmas period when nurseries are closed for up to two weeks.

### Feasibility and acceptability of NAP SACC UK

Overall, NAP SACC UK was implemented with good fidelity and was highly acceptable to key stakeholders. The two exceptions to this were i) the web-based parent element was not well used, and ii) one nursery did not fully implement the intervention (but participated in data collection). A summary of the programme implementation is shown in Table 1; we discuss each intervention element in turn below.

**Table 1:** Fidelity of implementation of NAP SACC UK

NAP SACC Partners							
Partner Number	Training	Top-up	Nursery Number	On-going Support			
				Email contacts (N)	Phone contacts (duration)		Meetings (duration)
Partner 14	✓	✓	8	5	1 (10 mins)		2 (180, 160 mins)
			9	5	1 (15 mins)		29,180, 160 mins)
Partner 25	✓	✓	1	1	1 (15 mins)		2 (150, 150 mins)
			5	2	3 (30, 5, 20 mins)		2 (150, 150 mins)
Partner 21	✓	✓	3	1	3 (10, 5, 10 mins)		3 (135, 90, 90 mins)
Partner 16	✓	✓	12	Nursery withdrew after first meeting to complete R&R			1 (unknown duration)
Nurseries							
Number	R&R completed		Workshops		Goals set (N)	Goals achieved (N)	Comments
	Base-line	Follow-up	PA (N staff)	Nutrition (N staff)			
1	✓	✓	8	5	8	7	Policy on PA not written
3	✓	✓	7	8	7	6	No data on one goal provided
5	✓	(✓^a^)	8	7	7	5	Policies on PA and nutrition not written
8	✓	✓	6	4	8	8	All goals achieved
9	(✓^a^)	✓	6	6	8	8	All goals achieved
12	✓	✘	n/a	n/a	n/a	n/a	Did not implement workshops or set goals
NAP SACC at Home Parent website							
Parent website launched					✓ April 2016		
Promotion of site (via flyers, mug and info sheet)					✓ April/May 2016		
Parents who logged onto website, N (%)					12 (14%)		
Parents who completed Healthy Habits form, N (%)					12 (14%)		
Parents who set ≥1 goal, N					7		
Texts/email sent, N					29		

^a^R&R forms completed, but not returned to the study team for analysis

### NAP SACK UK partner training

A training session for NAP SACC UK Partners was held in December 2015, facilitated by local experts. It provided an overview of the study, and training on how to support nurseries to review practice, set goals and implement changes. Partners found the training useful although noted *“it was a bit rushed”* (Partner_N12/16) and the sessions *“could have all been a bit longer”* (Partner_N1&5/25). The participants particularly appreciated the physical activity training, not having received formal training on this in their Health Visitor role. All four Partners undertook additional preparation outside of the training sessions to prepare for working with the nurseries, such as familiarising themselves with the literature, resources and documents provided by NAP SACC UK.

#### Review and Reflect self-assessment

All six intervention nurseries completed the Review and Reflect assessment at baseline, with five completing it again at follow-up. However, only three nurseries returned completed forms for both baseline and follow-up required for inclusion in the analysis (Tables 1 and 2). (In three cases, interviews confirmed the forms were completed but were subsequently misplaced). Self-reported summary scores increased from baseline to follow-up for all four areas, with the greatest improvement seen for ‘Physical Activity and Play’. Of the four individual items where no improvement was made, three related to policy development (see later section on goal setting). Across all items, there was a 9% increase and a mean difference of 0.26 (− 0.15, 0.67).

**Table 2:** Nursery self-reported Review & Reflect scores at baseline and follow-up (mean and SD)

Summary Scores	Baseline Mean (SD) (*n* = 3)	Follow up Mean (SD) (*n* = 3)
Child Nutrition and Oral Health Summary Score	3.07 (0.20)	3.24 (0.18)
Food provided	3.07 (0.20)	3.38 (0.21)
Beverages provided	3.44 (0.51)	3.56 (0.51)
Oral health	2.00 (0.33)	2.11 (0.51)
Feeding environment	3.24 (0.29)	3.51 (0.16)
Menus and variety	3 (1.00)	3 (0.00)
Nutrition education and professional development	2.29 (0.38)	2.95 (0.08)
Nutrition policy	2 (1.00)	2.33 (1.15)
Physical Activity and Play Summary Score	2.67 (0.29)	2.98 (0.15)
Time provided	2.78 (0.12)	3.22 (0.19)
Indoor play environment	3.33 (0.76)	3.50 (0.50)
Physical activity staff practices	3.08 (0.14)	3.25 (0.25)
Physical activity education and professional development	2.33 (0.31)	2.80 (0.35)
Physical activity policy	1 (0.00)	1 (0.00)
Outdoor Play and Learning Summary Score	2.57 (0.90)	2.71 (0.10)
Outdoor play	3.25 (0.75)	3.42 (0.52)
Outdoor physical environment	2.63 (0.45)	2.74 (0.53)
Outdoor play education and professional development	2 (0.88)	2.22 (0.51)
Outdoor play policy	1 (0.00)	1 (0.00)
Screen Time Summary Score	2.48 (0.33)	2.6 (0.23)
Screen time availability and staff practices	3.25 (0.50)	3.58 (0.52)
Screen time education	1.33 (0.58)	1.66 (0.58)
Screen time policy	1.0 (0.00)	1.0 (0.00)

The in-depth nature of the assessment meant it was time-consuming. Nonetheless, all managers found it a positive process with one commenting, “*it showed us what we are doing good and just the tweaks we needed to change that improved it”* (Manager_N9/4). The assessment was equally valued by the Partners as a way of getting managers to reflect critically on their current practice:

*“Certainly with them all pulling their menus out and it’s like, ‘Oh gosh, no, I didn’t think about that,’ and, ‘Oh yeah, look at that pattern, look at that. We’re offering too many biscuits there.’ Just gets them really thinking about it.”* (Partner_N8&9/14)

Some managers undertook their assessment independently while others completed it with their NAP SACC UK Partner. Partners who had done this felt it had aided their discussion, helping them understand the nursery context and set relevant goals. For one nursery in a more deprived area, the Partner noted this process *“gave me an oversight really, and also what restrictions they have from a financial point of view”* (Partner_N3/21).

#### Staff workshops

Two workshops (on physical activity and nutrition) were held for nursery staff in five of the six intervention nurseries. Workshops were conducted on Saturday mornings or after work, lasted between 2 and 3 h and were facilitated by local experts. The number of staff attending ranged from four to eight, with the NAP SACC UK Partner also often in attendance. Activities included presentations, quizzes, group discussions and games.

Nursery managers, staff and Partners were highly enthusiastic about the NAP SACC UK workshops, describing them as “*invaluable”* (Manager_N5/10), “*absolutely brilliant”* (Staff_N8/6) and *“inspiring”* (Partner_N8&9/14). Despite often occurring after work, participants reported feeling energised by the sessions: *“We all kind of came away like, ‘My gosh, I’ve got so much energy! ... It kind of re-inspired us.”* (Manager_N1/11). Observation and interview data highlighted the interactive tasks and the lively manner of the facilitators as being critical in engaging staff. The facilitators appeared skilled at adapting content and activities to the needs and interests of each group. One facilitator noticed and built upon the friendly competition emerging between groups during a quiz, while another facilitator was praised for helping staff overcome their shyness and enjoy the session. This was echoed by the nursery manager who commented:

*“*[The facilitator] *had like a funny character to him … it was kind of like we felt like we knew him … you just kind of felt at home or felt at ease. So you took on a lot more and you had a laugh and a joke, but you didn’t lose sight of where you were going.”* (Manager_N1/11)

Nursery staff appreciated the opportunity to reflect on their own practice, with one participant remarking the workshop had *“opened our eyes to think, ‘Actually, there are new ways of doing it.’*” (Staff_N8/6). Often changes suggested at the workshops were simple but perceived to be very powerful, for example, incorporating physical activity into story time or simply asking children ‘are you still hungry?’ instead of ‘do you want some more?’

While workshops received much praise, some issues were also identified. One Partner noted some staff were uncomfortable acting *“as if they were on CBEEBIES* [children’s television]*”* (Partner_N3/21) to encourage physical activity, but that this has been *“gently talked about* [in the workshop] *… and that’s something they’ve worked on”.* She added a staff member had subsequently been funded by the Local Authority to attend a storytelling workshop. Staff from another nursery in a deprived area reported feeling frustrated during workshops knowing they would be unable to implement many ideas due to budget restrictions: *“we were a little bit more deflated because we kind of thought, ‘Actually we know we can’t do that.’”* (Manager_N1/11).

While the workshops were offered free-of-charge, nurseries did incur costs in terms of staff time-off-in-lieu, ranging from £478–£691 (mean £610) per nursery. However, no manager raised this incurred cost as being problematic during interviews. When asked if they would have been prepared to pay for the workshops, most were equivocal suggesting they may have done, but would have limited the number of staff attending.

#### Goal setting

Goals were set in five of the six intervention nurseries with the majority achieved within the intervention period (see Table 1); those that required more effort on the part of the manager (e.g. writing new policies) had not yet been achieved. Many goals were easy and simple to implement, which one Partner felt was key to engaging nurseries: *“They’re just small, inexpensive goal setting … I thought that was practical, not too big a jump for the nurseries.”* (Partner_N12/16).

Three nurseries set goals to modify their menus by, for example, providing more fruit, removing biscuits as snacks and offering oily fish once a week. Others modified portion sizes, with one manager commenting *“we’re not over feeding them anymore”* (Manager_N3/1). One nursery let staff eat for free to encourage them to role model positive eating behaviours, leading one staff member to comment, *“that’s such a benefit to those children … to see I am eating my vegetables, and they can copy me and copy that behaviour”* (Staff_N3/2). Four nurseries set goals to provide nutritional information to parents via parents’ evenings, notice boards, leaflets and websites. One manager felt the nursery’s involvement with NAP SACC UK provided the legitimacy and *“clout”* needed to raise nutrition topics with parents (Manager_N5/10), while another described how they now included a healthy lunchbox guide in their welcome pack as standard (Manager _N1/11).

Physical activity goals focused on three areas: writing policies; providing information to parents; and changing staff practice and/or use of space or equipment to promote physical activity. Only one nursery had devised a physical activity policy, while two other nurseries selected this goal but time constraints limited its achievement. It was suggested that providing template policies would help nurseries achieve this goal. Interview participants tended to focus on the new ways they had found to increase physical activity throughout the day. One nursery had improved their outside space, while another moved from having set garden times to allowing children to choose to play indoors or out. Importantly, the intervention allowed staff to reflect on their own practice and gave them permission to focus on physical activity:

*“It was just quite nice to see the staff actually relaxing a bit more and not worried about, ‘Oh, we need to go to do that next’ … And for the children it was, ‘Oh, the adults do it too, they have fun, they play the spot game, they chase us round.’”* (Manager_N1/11)

Though most goals had been met, it became apparent that some nurseries had chosen not to set certain goals, knowing they would be unachievable. For example, one Partner had not suggested writing new policies in one nursery as it was part of a national chain and “*would have gone through a lot of red tape and wouldn’t have been achieved within the timeframe”* (Partner_N8&9/14). Another nursery discussed, but did not set, tooth brushing as a goal as they did not have space to accommodate the extra movement of children and staff.

Budgetary constraints were a significant barrier in one nursery operating in a very deprived area. The manager wanted to reduce the amount of bread offered at teatime and provide healthier alternatives but the nursery owners were unable or unwilling to increase the budget. As the manager explained, *“when you can’t exceed 40p for a loaf of bread, how could you possibly buy a thing of wraps and something to fill the wraps?”* (Manager_N1/11).

#### On-going partner support

Partners and managers from the five nurseries that fully implemented the intervention all met in person at least twice: first, to review practice and set goals, and second, to assess progress. Additional support was provided via phone calls and emails. Managers and staff reported finding this on-going contact with Partners useful. One manager appreciated the Partner’s outside perspective and found the positive feedback she received very motivating. Others valued the opportunity to develop a relationship with another child health professional: *“It’s nice that I’ve got that professional relationship now with our* [Partner]. *So I know that I can call her any time and she’ll come down even after the study has finished”* (Staff_N3/2). However one Partner appeared uncomfortable in providing this support to busy nursery managers, admitting *“I do feel like I’m harassing them”* (Partner_N1&5/25). Though she provided guidance and signposting to relevant resources she explained: *“I don’t think I really got to that point where … they were ringing me up saying, “Oh, how can we do this?” I didn’t really have that relationship with them.”* (Partner_N1&5/25).

Within NAP SACC UK, the role of Partner was taken on by local Health Visitors who were deemed to be similar in skills and professional background to the child-care health Partners used in NAP SACC in the United States [15]. For this study, Partners were employed by NAP SACC UK, with this work occurring outside of their normal Health Visiting role and contracted hours. Partners felt taking on the NAP SACC role was appropriate as Health Visitors are the *“only consistent professionals that are seeing that age group, outside of the nurseries”* (Partner_N3/21). While one Partner queried the necessity of using Health Visitors for this role, others felt the *“in-depth”* and *“holistic*” approach of the Health Visitor was important and their professional status appeared to be valued by nursery managers. However, all Partners raised concerns regarding the capacity of Health Visitors to deliver this role in any subsequent roll-out, noting that their existing workload was so great that *“if you put this on health visitors to do routinely, I think it just wouldn’t get done.”* (Partner_N12/16). Family Support Workers and Nursery Nurses were suggested as possible alternatives for the Partner role, although it was noted that these staff have similar capacity constraints and lower levels of training than Health Visitors.

#### Web-based parent element

The web-based parent element of NAP SACC UK was not well-used. The site was launched in April 2016 and promoted to parents at intervention nurseries via information sheets, flyers and a promotional mug. They were also offered a free family swimming voucher if they completed the ‘Healthy Habits’ questionnaire and set a goal. However, only 12 parents (14%) logged onto the website and only seven set a goal. All 12 parents were emailed to encourage them to set a (further) goal, but none did so.

Five of the ten intervention parents interviewed had used the website. Of the remaining five, three had been unaware of it, while one did not “*get around to looking at it”* (Parent_32) and the other explained, *“it hasn’t been a priority really”* (Parent_19).

Those using the website found logging on, completing the ‘Healthy Habits’ questionnaire and setting goals easy to do. However, one parent questioned the usefulness of the site explaining *“I think probably if I wanted advice or anything I would turn to the internet anyway and good old Google for ideas”* (Parent_22). Two parents discussed setting goals but neither felt it had made much difference to their interactions with their child. One tried to make fruit and vegetables more appealing by arranging them in the shape of a butterfly but *“the novelty wore off after about a minute”* (Parent_29). The other tried to be more mindful of what she was giving her child to eat but this had tailed off over the summer holidays while they were out of their normal routine. Text messages sent to two parents were not well received because they were too generic and *“nothing that I didn’t know”* (Parent_29).

#### Withdrawal from the intervention: nursery 12

As noted above, one nursery (12) did not fully implement the intervention; the manager completed the self-assessment with the Partner, but no goals were set and no workshops held. The primary reason for this withdrawal was that the staff were unwilling to attend the required out-of-hours workshops because they had recently completed several weeks of mandatory out-of-hours first aid training. Importantly, therefore, it was the timing rather than the out-of-hours nature of the workshops that was problematic.

A letter of agreement setting out what participation in the study would involve (including the two workshops) had been sent to all nurseries at recruitment. However in Nursery 12 this letter had been signed by the deputy manager; it became clear during the manager interview that she had been unaware of this requirement. Although alternative solutions were presented (including offering workshops during the day), none was acceptable to the manager. This led the nursery to withdraw from the intervention, but they participated in follow-up data collection.

### Staff and parent mediators

The NAP SACC UK intervention draws on components of social cognitive theory, framed within a socioecological framework [21, 25]. The intervention logic model therefore theorised that changes in knowledge, motivation and self-efficacy in nursery staff and parents would be important mediators through which the intervention impacted on key child health outcomes such as BMI (see Additional file 1 and [26]). For nursery staff receiving the intervention, there were small increases in all mediators from baseline to follow-up, except for nutrition self-efficacy (Table 3). In control nurseries, there were small decreases in all mediators apart from physical activity knowledge and self-efficacy. Intervention parents reported small increases in all mediators but physical activity knowledge; in control parents, all three physical activity mediators increased slightly, with decreases or minimal change in nutrition mediators (Table 4).

**Table 3:** Nursery staff reported mediators of knowledge, self-efficacy and motivation (mean and SD)

	Intervention				Control			
	Baseline		Follow-up		Baseline		Follow-up	
	n	Mean (SD)	n	Mean (SD)	n	Mean (SD)	n	Mean (SD)
Physical activity, play & sedentary time								
Knowledge	16	4.19 (1.64)	12	5.08 (0.90)	15	4.07 (1.28)	11	4.64 (1.69)
Motivation	15	4.54 (0.45)	11	4.76 (0.32)	15	4.45 (0.84)	11	4.36 (0.76)
Self-efficacy	16	4.43 (0.47)	12	4.58 (0.39)	15	4.46 (0.57)	10	4.54 (0.43)
Nutrition & oral health								
Knowledge	16	10.88 (3.30)	12	12.09 (2.02)	15	11.00 (1.13)	11	10.20 (2.66)
Motivation	15	4.47 (0.50)	11	4.66 (0.38)	15	4.58 (0.51)	10	4.28 (0.89)
Self-efficacy	16	4.36 (0.36)	10	4.35 (0.54)	15	4.58 (0.29)	10	4.38 (0.49)

Note that only a proportion of nursery staff responded at both time points; this table includes data from staff who responded at either one or both time points

**Table 4:** Parent reported mediators of knowledge, self-efficacy and motivation (mean and SD)

	Intervention				Control			
	Baseline		Follow-up		Baseline		Follow-up	
	n	Mean (SD)	n	Mean (SD)	n	Mean (SD)	n	Mean (SD)
Physical activity, play & sedentary time								
Knowledge	32	4.00 (1.30)	32	3.78 (1.41)	41	4.15 (1.15)	41	4.56 (1.23)
Motivation	32	4.12 (0.52)	32	4.33 (0.53)	40	4.24 (0.60)	40	4.29 (0.49)
Self-efficacy	32	4.20 (0.51)	32	4.28 (0.58)	41	4.39 (0.52)	41	4.43 (0.47)
Nutrition & oral health								
Knowledge	32	9.97 (2.24)	32	10.69 (1.84)	41	10.34 (2.16)	41	10.22 (2.12)
Motivation	32	4.25 (0.66)	32	4.39 (0.55)	41	4.32 (0.55)	41	4.34 (0.51)
Self-efficacy	32	4.37 (0.49)	32	4.49 (0.54)	40	4.52 (0.45)	40	4.49 (0.47)

### Acceptability of trial design and methods

As few interventions have been conducted in Early Years settings, we assessed the feasibility and acceptability of collecting a range of data from staff, parents and children.

#### Child height, weight and accelerometry

Staff reported few problems with regards to weighing and measuring children in the nursery setting, noting that most children enjoyed it. As each child being measured needed to be accompanied by a staff member, there was some disruption, but this was managed by providing extra staff or by using a private, quiet area in the same room. None of the parents interviewed reported any reservations about having their child weighed or measured.

The majority of children enjoyed wearing the accelerometry belts according to staff and parents, particularly because study staff presented them as ‘superhero belts’. Staff found it easier than parents to get children to wear the belts because other children in the class were also doing so. However, both staff and parents found it more challenging to persuade children to wear them at follow-up because the novelty had worn off: 88% children wore belts at baseline vs. 74% at follow-up. Few problems were reported, but included: belts being uncomfortable; forgetting to wear them; misplacing or mixing up belts; and having to move belts back to the correct position often. One manager reported a mother was concerned her child might put the belt around her neck, but otherwise there were no comments about safety.

#### Observations in nurseries: EPAO and CADET

Two sets of observations were carried out in each nursery: a comprehensive whole-day assessment of the nursery environment (EPAO); and mealtime observations to record what children were eating (CADET). In general, few issues were reported with either observation, with one manager explaining the NAP SACC UK observers “*just sort of melted to the background ... You hardly know they’re there”* (Manager_N5/10)*.* However, one staff member mentioned the logistical difficulties of accommodating two extra adults in their very limited space, while another talked about the inconvenience of letting the observers in and out of the nursery every meal time.

There were mixed comments regarding how staff felt about being observed. Several noted they were used to being observed (for example during official inspections) and were unconcerned, while others reported finding it *“a little strange”* (Manager_N8/7) or *“uncomfortable”* (Manager_N10/8). Equally, some managers felt their staff had been unaffected by being observed, while two explained their staff may have changed their behaviour somewhat: *“I wouldn’t say it changed what they did, but it may have changed the way they did it a little bit.”* (Manager_N8/7).

NAP SACC staff were often praised by managers regarding the various data collection processes. Managers appreciated the study staff being flexible to fit in with the usual nursery schedule and minimise disruption as far as possible, as well as their friendly and reassuring interactions with the children. Due to the logistics of the study, different NAP SACC UK staff attended different nurseries at each data collection point, leading some managers to suggest they would have preferred a more consistent relationship with study staff allowing them to build rapport with nursery staff and children.

#### Parent questionnaires and food diaries

Parents were sent a questionnaire at baseline and follow-up, including questions on the child’s quality of life (PedsQL), spending on food and physical activities, healthcare usage and mediators such as knowledge, self-efficacy and motivation. Most parents reported the questionnaires to be *“straightforward”,* albeit time consuming. Equally, most completing the food diary found it unproblematic. A minority however reported finding it confusing and were unclear as to which days/meals they needed to complete or which category certain foods fell into. Nursery staff confirmed some parents appeared unsure how to complete the diary, but because they had not seen the instructions given to parents they found it difficult to help.

## Discussion

NAP SACC UK was implemented with high fidelity, with two exceptions. First, the out-of-hours workshops were unacceptable to one nursery, following shortly after several weeks of mandatory out-of-hours training. Second, the home component for parents was not well used and should be removed from any subsequent trial. In addition, recruitment was more challenging in Gloucestershire where other nursery-based initiatives were already running. Barring these issues, the intervention was well-received by parents, nursery staff and Partners. Nursery staff were motivated by the workshops, and managers and Partners reviewed current practices and set practical, realistic goals which for the most part were achieved. Importantly, nursery managers were willing to bear the indirect costs of the workshops in terms of staff time-off-in-lieu, which averaged £610. Collection of a range of data from children, staff and parents was acceptable and feasible. Well-trained staff who were able to build rapport with children and staff and willing to be flexible to accommodate nursery schedules were critical in facilitating the data collection.

### Mechanisms of impact

The NAP SACC UK logic model [20] suggests the intervention works through changes to staff and parents’ knowledge, motivation and perceptions of self-efficacy to improve children’s nutrition and levels of physical activity. Though this study was designed to assess feasibility rather than measure changes in these mediators, it is encouraging that knowledge and motivation increased in staff in intervention nurseries in comparison to controls. This complements qualitative findings, suggesting staff valued the workshops, felt energised and motivated by them and were able to achieve the majority of their goals. There was little change in parents’ mediators but given the small sample size and lack of engagement in the web-based parent component, this is unsurprising.

Qualitative process data provides further insights into the mechanisms by which the intervention promoted change. Being given the opportunity to review their own policies and practice was clearly valued by the managers, and something difficult to do in a busy nursery day without the support of the intervention. It also appeared that being given ‘permission’ to focus on nutrition and physical activity was important for staff to fully engage in the intervention process. This carving out of reflexive space in a busy nursery setting may therefore be an important mechanism by which change is initiated and sustained.

Most goals set were simple and easy to achieve, and this may have been important in engaging staff. One Partner felt these ‘quick wins’ helped staff feel the intervention was manageable and maintained their positive feeling towards the changes. Equally, reviewing practices with someone external to the nursery (the Partner) appeared important in holding managers accountable, particularly because Partners were valued child health professionals. However, it is important to acknowledge some goals were not set specifically because the managers and Partners knew they would be difficult to achieve. Ideally, NAP SACC UK would become embedded into nursery practice allowing for on-going reflection, goal setting and improvement. Yet it is unclear if and how this would work in practice once the easiest changes had been made and managers had to tackle more challenging and complex issue. Equally, maintenance of new practices through a sense of shared responsibility and vision may prove challenging in the context of high staff turnover or other contextual issues (e.g. changes in food supplier).

Finally, the workshops appeared one of the most important aspects of the intervention in creating a shared enthusiasm and vision for change within nursery staff. The content of the workshops was highly valued by staff, particularly the physical activity session which for many staff was a relatively novel topic. However, the facilitation style and personality of the workshop leaders was as (if not more) important than the content in engaging staff and creating ‘buy in’ from those interacting with the children on a daily basis. Selecting facilitators with the right qualities to deliver workshops will likely be important to success in any future trial or roll-out.

While managers were willing to absorb the staff overtime costs, most would have limited staff participation to only one or two members of staff had they been charged for the workshops. However, the shared enthusiasm created by the workshops appeared an important mechanism by which change was created in the nurseries and should be protected in any subsequent intervention roll-out.

### Contextual factors

Critical to NAP SACC UK process evaluation was exploration of why Nursery 12 did not fully implement the intervention as, on face value, this might suggest the intervention was unacceptable to nursery staff. In fact, circumstances specific to this nursery led to its withdrawal. First, nursery staff had recently completed several out-of-hours training sessions making further out-of-hours workshops unacceptable. Second, the letter of agreement was signed by the deputy manager, meaning the manager was unaware of all the study requirements. These local issues suggest there is nothing inherently flawed in the NAP SACC UK intervention – not least because the five other nurseries implemented it with high fidelity. Nonetheless, these contextual factors provided important learning for future trials, particularly in terms of the scheduling of the intervention and communication of what it entails.

The process evaluation also highlighted potential issues with the sustainability of NAP SACC UK in its current form. NAP SACC UK Partners were local Health Visitors well-suited to the role, and appreciated by managers because of their professional background. However, all Partners raised concerns about the capacity of Health Visitors to take on this role in the context of staff shortages and ever-expanding caseloads. A future full-scale trial and potential roll-out of the programme will need to consider carefully how the Partner role can be managed to ensure sustainability while maintaining the integrity of the intervention.

The process evaluation embedded within this feasibility study provided useful insights into the intervention’s mechanisms of change and the importance of context. There is a paucity of high quality process contextual data relevant to early years settings. Of 16 RCTs [11–14, 27–40] targeting childcare settings (in America, Europe and Australia), eight collected no (or very minimal) process data and only one [13] conducted a comprehensive process evaluation combining qualitative and quantitative methods. Our findings thus make a valuable contribution to the development of public health interventions in these settings.

## Conclusion

NAP SACC UK has proved feasible to implement and acceptable to key stakeholders, albeit with the removal of the parent-based element. There is an urgent need for robustly-evaluated obesity interventions in early years settings. A full-scale trial to test NAP SACC UK’s effectiveness on key health outcomes is warranted and a definitive trial will start in July 2019.

## Additional file

Additional file 1: NAP SACC UK Logic Model. (PDF 427 kb)

## Data Availability

The datasets used and/or analysed during the current study are available from the corresponding author on reasonable request.

## Abbreviations

BMI: Body Mass Index
CADET: Child and Diet Evaluation Tool
EPAO: Environment Policy Assessment and Observation
MRC: Medical Research Council
NAP SACC: Nutrition and Physical Activity Self-Assessment for Childcare
PA: Physical Activity
PedsQL: Pediatric Quality of Life
R&R: Review and Reflect self assessment
RCT: Randomised Controlled Trial
SD: Standard Deviation
UK: United Kingdom
USA: United States of America
zBMI: Body Mass Index, standardised for age and sex

## References

[1] World Health Organization: Facts and figures on childhood obesity. http://www.who.int/end-childhood-obesity/facts/en/. Accessed 30 Apr 2018.

[2] Simmonds M, Llewellyn A, Owen C, Woolacott N (2016). Predicting adult obesity from childhood obesity: a systematic review and meta-analysis. Obes Rev.

[3] NHS Digital: National Child Measurement Programme England, 2016/17 school year. 2017. https://digital.nhs.uk/catalogue/PUB30113 Accessed 30 Apr 2018 2017.

[4] Scholes S: Health survey for England 2015: physical activity in children. NHS digital 2016. https://files.digital.nhs.uk/publicationimport/pub22xxx/pub22610/hse2015-child-phy-act.pdf. Accessed 30 Apr 2018.

[5] Public Health England & Food Standards Agency: National Diet and Nutrition Survey Results from Years 7 and 8 (combined) of the Rolling Programme (2014/2015–2015/2016). 2018.

[6] Department for Education: Statistical First Release 29/2017: Provision for children under five years of age in England: June 2017. https://www.gov.uk/government/uploads/system/uploads/attachment_data/file/622632/SFR29_2017_Text.pdf. Accessed 30 Apr 2018. 2017.

[7] Department for Education: Childcare bill: policy statement. https://assets.publishing.service.gov.uk/government/uploads/system/uploads/attachment_data/file/482517/Childcare_Bill_Policy_Statement_12.03.2015.pdf. Accessed 30 Apr 2018. 2015.

[8] Department of Health and Social Care: UK Physical Activity Guidelines. Fact sheet 2: early years (under 5s capable of walking). https://assets.publishing.service.gov.uk/government/uploads/system/uploads/attachment_data/file/213738/dh_128143.pdf. Accessed 30 Apr 2018.

[9] Children's food trust. eat better start better: voluntary food and drink guidelines for early years settings in England. London: Children's food trust. Available from: https://www.foundationyears.org.uk/wp-content/uploads/2017/11/Eat-Better-Start-Better1.pdf. Accessed 19 Apr 2019; 2017.

[10] HM Government: Statutory Instruments 2014 No. 1603. Education, England. The Requirements for School Food Regulation. http://www.legislation.gov.uk/uksi/2014/1603/pdfs/uksi_20141603_en.pdf. Accesssed on 30 Apr 2018. 2014.

[11] O’Dwyer MV, Fairclough SJ, Knowles Z, Stratton G (2012). Effect of a family focused active play intervention on sedentary time and physical activity in preschool children. Int J Behav Nutr Phys Act.

[12] Foulkes JD, Knowles Z, Fairclough S, Stratton G, O’Dwyer M, Ridgers N, Foweather L (2017). Effect of a 6-week active play intervention on fundamental movement skill competence of preschool children:a cluster randomized controlled trial. Percept Mot Skills.

[13] Watt R (2012). Exploratory and developmental trial of a family centred nutrition intervention delivered in Children's Centres.

[14] Reilly JJ, Kelly L, Montgomery C, Williamson A, Fisher A, McColl JH, Lo Conte R, Paton JY, Grant S (2006). Physical activity to prevent obesity in young children: cluster randomised controlled trial. Bmj.

[15] Ward DS, Benjamin SE, Ammerman AS, Ball SC, Neelon BH, Bangdiwala SI (2008). Nutrition and physical activity in child care: results from an environmental intervention. Am J Prev Med.

[16] Alkon A, Crowley AA, Neelon SE, Hill S, Pan Y, Nguyen V, Rose R, Savage E, Forestieri N, Shipman L (2014). Nutrition and physical activity randomized control trial in child care centers improves knowledge, policies, and children's body mass index. BMC Public Health.

[17] Bonis M, Loftin M, Ward D, Tseng TS, Clesi A, Sothern M (2014). Improving physical activity in daycare interventions. Child Obes.

[18] Department for Education: Statutory Framework for the Early Years Foundation Stage: Setting the Standards for Learning, Development and Care for Children from Birth to Five. https://www.foundationyears.org.uk/files/2017/03/EYFS_STATUTORY_FRAMEWORK_2017.pdf: Department for Education; 2017.

[19] Public Health England: Child Oral Health: Applying All Our Health. Available from https://www.gov.uk/government/publications/child-oral-health-applying-all-our-health/child-oral-health-applying-all-our-health. Accessed 1 May 2018. 2018.

[20] Kipping R, Langford R, Brockman R, Wells S, Metcalfe C, Papadaki A, White J, Hollingworth W, Moore L, Ward D et al. Physical activity, oral health and nutrition environmental nursery and web-based home intervention for 2–4 year olds: NAP SACC UK feasibility cluster RCT. Public Health Research; 2019. In Press. 10.1186/s12889-019-7102-9 PMC6609387 31269926

[21] Kipping R, Jago R, Metcalfe C, White J, Papadaki A, Campbell R, Hollingworth W, Ward D, Wells S, Brockman R (2016). NAP SACC UK: protocol for a feasibility cluster randomised controlled trial in nurseries and at home to increase physical activity and healthy eating in children aged 2–4 years. BMJ Open.

[22] Dias K, White J, Metcalfe C, Kipping R, Papadaki A, Jago R: Acceptability, internal consistency and test-retest reliability of scales to assess parental and nursery staff’s self-efficacy, motivation and knowledge in relation to preschoolers’ nutrition, oral health and physical activity. Public Health Nutrition In press. 10.1017/S1368980018004111 PMC6520240 30761972

[23] Braun V, Clarke V (2006). Using thematic analysis in psychology. Qual Res Psychol.

[24] The Bristol Standard. Available from https://www.bristolearlyyears.org.uk/the-bristol-standard/, Accessed 21 Jan 2019.

[25] Benjamin SE, Ammerman A, Sommers J, Dodds J, Neelon B, Ward DS (2007). Nutrition and physical activity self-assessment for child care (NAP SACC): results from a pilot intervention. J Nutr Educ Behav.

[26] Dias K, White J, Metcalfe C, Kipping R, Papadaki A, Jago R. Acceptability, internal consistency and test–retest reliability of scales to assess parental and nursery staff’s self-efficacy, motivation and knowledge in relation to pre-school children’s nutrition, oral health and physical activity. Public Health Nutr. 2019:1–9. 10.1017/S1368980018004111 PMC6520240 30761972

[27] Alhassan S, Sirard JR, Robinson TN (2007). The effects of increasing outdoor play time on physical activity in Latino preschool children. Int J Pediatr Obes.

[28] Binkley T, Specker B (2004). Increased periosteal circumference remains present 12 months after an exercise intervention in preschool children. Bone.

[29] Dennison BA, Russo TJ, Burdick PA, Jenkins PL (2004). An intervention to reduce television viewing by preschool children. Arch Pediatr Adolesc Med.

[30] Fitzgibbon ML, Stolley MR, Schiffer LA, Braunschweig CL, Gomez SL, Horn L, Dyer AR (2011). Hip-hop to health Jr. obesity prevention effectiveness trial: Postintervention results. Obesity.

[31] Trost SG, Fees B, Dzewaltowski D (2008). Feasibility and efficacy of a “move and learn” physical activity curriculum in preschool children. J Phys Act Health.

[32] Hardy L, King L, Kelly B, Farrell L, Howlett S (2010). Munch and move: evaluation of a preschool healthy eating and movement skill program. Int J Behav Nutr Phys Act.

[33] De Bock F, Breitenstein L, Fischer JE (2012). Positive impact of a pre-school-based nutritional intervention on children’s fruit and vegetable intake: results of a cluster-randomized trial. Public Health Nutr.

[34] Bayer O, von Kries R, Strauss A, Mitschek C, Toschke AM, Hose A, Koletzko BV (2009). Short-and mid-term effects of a setting based prevention program to reduce obesity risk factors in children: a cluster-randomized trial. Clin Nutr.

[35] Manios Y, Grammatikaki E, Androutsos O, Chinapaw M, Gibson E, Buijs G, Iotova V, Socha P, Annemans L, Wildgruber A (2012). A systematic approach for the development of a kindergarten-based intervention for the prevention of obesity in preschool age children: the ToyBox-study. Obes Rev.

[36] Natale R, Ludwig D, Sardinas K, Change Martinez C, Palenzuela J, M S SM (2017). NIFA poster abstract healthy caregivers healthy children phase 2 (HC2): relationship between childcare center nutrition and physical activity environment and child body mass index over one school year. J Nutr Educ Behav.

[37] Roth K, Kriemler S, Lehmacher W, Ruf KC, Graf C, Hebestreit H (2015). Effects of a physical activity intervention in preschool children. Med Sci Sports Exerc.

[38] Roth K, Mauer S, Obinger M, Ruf KC, Graf C, Kriemler S, Lenz D, Lehmacher W, Hebestreit H (2010). Prevention through activity in kindergarten trial (PAKT): a cluster randomised controlled trial to assess the effects of an activity intervention in preschool children. BMC Public Health.

[39] Pate RR, Brown WH, Pfeiffer KA, Howie EK, Saunders RP, Addy CL, Dowda M (2016). An intervention to increase physical activity in children: a randomized controlled trial with 4-year-olds in preschools. Am J Prev Med.

[40] De Craemer M, De Decker E, Verloigne M, De Bourdeaudhuij I, Manios Y, Cardon G (2014). The effect of a kindergarten-based, family-involved intervention on objectively measured physical activity in Belgian preschool boys and girls of high and low SES: the ToyBox-study. Int J Behav Nutr Phys Act.

