# Supplementary material for: A physical activity, nutrition and oral health intervention in nursery settings: process evaluation of the NAP SACC UK feasibility cluster RCT
Source: BMC Public Health. 2019 Jul 3;19:865. doi: 10.1186/s12889-019-7102-9 (PMC6609387; doi:10.1186/s12889-019-7102-9)
Supplement: Supplementary file 1 — NAP SACC UK Logic Model. (PDF 427 kb) [file 12889_2019_7102_MOESM1_ESM.pdf]

Logic Model: NAP SACC UK

Household and nursery environment characteristics

Socio-demographic factors for the child and family: area-level deprivation (IMD Score using home postcode); gender; ethnicity

Nursery environment factors self-reported by nursery: nursery policy to promote healthy eating and physical activity and reduce sedentary behaviours; external initiatives to promote healthy eating and physical activity and/or reduce sedentary behaviour

Nursery factors reported on national website: Ofsted school performance factors

Intervention

NURSERY: 'Review and reflect' against best practice with regular targeted assistance from a NAP SACC UK Partner to undertake goal setting and action planning to change the environment, policies and practices:

Nutrition	-Fruit and veg<br>-High-fat foods<br>-Beverages<br>-Meals and snacks<br>-Menu and variety<br>-Food items outside of regular meals and snacks<br>-Supporting healthy eating<br>-Oral health promotion<br>-Nutrition education for children, parents & staff<br>-Nutrition policy
Physical activity	-Active play and inactive time<br>-Screen use and viewing<br>-Play environment<br>-Supporting physical activity<br>-Physical activity education for children, parents and staff<br>-Physical activity policy

HOME: 'NAP SACC at Home' component for parents to review nutrition, physical activity, sedentary, oral health and sleep behaviours at home with ideas for changes at home supported by post, text and email reminders.

Mediators (method of measurement in brackets)

Knowledge	Nursery staff/parents increased knowledge of best practice about nutrition and physical activity (process evaluation)
	Nursery staff/parents increased knowledge of health benefits of nutrition, physical activity, decreasing sedentary time and health risks for children from excess weight (process evaluation)
Self-efficacy and motivation	Nursery staff/parents strengthened self-efficacy and internal motivation for improving children and staff health, improving children's nutrition and physical activity (self-efficacy and motivation measures)

Outcomes (method of measurement in brackets)

Nursery environment factors assessed by Environmental Policy and Observation Assessment

Physical activity and diet behaviours	Increased moderate and vigorous physical activity (accelerometry)
	Decreased sedentary time (accelerometry)
	Increased fruit and vegetables consumed per day (CADET)
	Decreased unhealthy snacks, high fat foods and high energy drinks consumed per day (CADET)
	Decreased screen viewing (questions added to CADET)
Obesity-related	Reduced body mass index (if overweight or obese) (zBMI)
	Decreased prevalence of overweight and obesity (zBMI and IOTF/UK1990 thresholds)

Guidance and policy context

Eat Better Start Better; Change4Life; Food and Health Guidelines for early years and childcare settings; Start Active, Stay Active: a report on physical activity for health from the four home countries' Chief Medical Officers
